# Neural signature of error processing in major depression

**DOI:** 10.1007/s00406-021-01238-y

**Published:** 2021-02-17

**Authors:** Kathrin Malejko, Stefan Hafner, Paul L. Plener, Martina Bonenberger, Georg Groen, Birgit Abler, Heiko Graf

**Affiliations:** 1 Department of Psychiatry and Psychotherapy III, Ulm University Hospital, Leimgrubenweg 12-14, Ulm, Germany; 2 Department of Child and Adolescent Psychiatry and Psychotherapy, Ulm University Hospital, Ulm, Germany; 3 Department of Child and Adolescent Psychiatry, Medical University of Vienna, Vienna, Austria

**Keywords:** Error processing, Commission errors, Major depression, Go/NoGo

## Abstract

The clinical presentation of major depression (MD) is heterogenous and comprises various affective and cognitive symptoms including an increased sensitivity to errors. Various electrophysiological but only few functional magnetic resonance imaging (fMRI) studies investigated neural error processing in MD with inconsistent findings. Thus, reliable evidence regarding neural signatures of error processing in patients with current MD is limited despite its potential relevance as viable neurobiological marker of psychopathology. We therefore investigated a sample of 16 young adult female patients with current MD and 17 healthy controls (HC). During fMRI, we used an established Erikson-flanker Go/NoGo-paradigm and focused on neural alterations during errors of commission. In the absence of significant differences in rates of errors of commission in MD compared to HC, we observed significantly (*p* < 0.05, FWE-corrected on cluster level) enhanced neural activations of the dorsal anterior cingulate cortex (dACC) and the pre-supplementary motor area (pre-SMA) in MD relative to HC and thus, in brain regions consistently associated to neural error processing and corresponding behavioral adjustments. Considering comparable task performance, in particular similar commission error rates in MD and HC, our results support the evidence regarding an enhanced responsivity of neural error detection mechanisms in MD as a potential neural signature of increased negative feedback sensitivity as one of the core psychopathological features of this disorder.

**Supplementary Information:**

The online version contains supplementary material available at 10.1007/s00406-021-01238-y.

## Introduction

Major depression (MD) is one of the most commonly diagnosed mental disorders in first world countries affecting more than 264 million people worldwide [1]. The disorder is characterized by core symptoms of persistent feelings of depressed mood or loss of interest, and is diagnosed by behavioral observations according to the diagnostic criteria defined in the DSM-5 [2]. However, it is well known that the clinical presentation of MD is heterogenous and symptoms comprise a culmination of various affective and cognitive deficits. In particular, converging evidence suggests valence-specific maladaptions in MD, such as an excessive sensitivity to negative feedback and errors of commission [3–5], i.e. differences between intended and executed responses. This increased sensitivity may act as a crucial psychological factor in the development and maintenance of this disorder.

Electrophysiological studies have intensively investigated neurofunctional signatures of error processing. These studies inferred the existence of a generic, high-level human error processing system from the error-related negativity (ERN), a negative deflection in an event-related potential component elicited about 50–100 ms after erroneous responses [6–10]. The anterior cingulate cortex (ACC) and adjacent pre-supplementary motor area (pre-SMA) were identified as a core system in error processing and as neural generator of the ERN [11–14]. In addition, functional magnetic resonance imaging (fMRI) studies reliably demonstrated the involvement of the inferior frontal cortex, encompassing the frontal operculum and the anterior insula in error processing [12, 15–18].

Various electrophysiological studies have also investigated neural error processing in MD, but revealed heterogenous findings regarding the amplitude of the ERN. In particular, studies using experimental tasks devoid of incentive manipulations, observed an elevated ERN in middle-aged and elderly patients with MD [19–21]. Other studies also demonstrated an increase in ERN amplitudes but only for errors after erroneous trials [22, 23]. However, no differences in ERN amplitudes in MD compared to healthy controls (HC) were observed within the context of reward [20], and other studies even found an attenuated ERN-amplitude in young patients with MD [24], or in severely depressed patients during commission errors following erroneous trials [25, 26].

Only few fMRI studies investigated the neurofunctional signature of error processing in MD so far. One fMRI study investigated neural error processing in patients with remitted MD compared to HC and demonstrated neural hypoactivity within the rostral ACC (rACC) and the dorsomedial prefrontal cortex (dmPFC) during commission errors [27]. A more recent fMRI-study in patients with remitted MD associated an attenuated neural activation within the middle frontal gyrus during commission errors with higher risk for depression recurrence [28]. Another fMRI-study mainly focused on neural activations of inhibitory control and corresponding treatment response, but investigated neural alterations of error processing in MD with a contextual parametric Go/NoGo-paradigm with three separate levels [29]. This study demonstrated an increase in neural activations within the medial frontal gyrus and the precuneus during errors of commission in MD relative to HC.

Of note, the examination of neural responses to errors is thought to be a viable neurobiological marker of psychopathology [30–33] that holds promise towards improving diagnostic procedures and treatment outcome or monitoring. Furthermore, there is an ongoing search for neurobiological markers, that could have the potential to serve as predictors for pharmacological treatment response in tailoring individual pharmacotherapy to patients´ needs. Considering the inconsistent findings regarding neural signatures of error processing in MD that presumably owe to different symptom state, symptom severity and experimental tasks, there is an inevitable need for replication and validation of neural responses of error processing in independent samples before these potential biomarkers can be translated into clinical practice. We therefore investigated a sample of patients with current major depression and non-depressive, healthy controls. During fMRI, we used an established modified Erikson-flanker Go/NoGo-paradigm without incentive manipulations, that has been shown to reliably elicit neural responses in brain areas corresponding to commission errors and error processing [34–37]. Based on previous electrophysiological and neuroimaging studies using non-rewarding tasks [19–21, 29], we expected an increased neural response in MD of brain regions previously related to error processing, and in particular of the ACC.

## Methods

### Subjects

As part of a broader research project with different experiments of which findings are reported elsewhere [38, 39], we investigated a total of 33 young adult female participants aged 18–38 years. Of those, 16 patients were diagnosed with MD whereas 17 healthy participants (HC) served as control group with no current or lifetime psychiatric diagnosis. Participants in the MD- and HC-group were matched for their highest degree of education. Of note, patients with MD were on average about 5 years older than HC (Table 1). Two subjects in the MD-group also met the diagnostic criteria of dysthymia according to DSM-5. One further subject in the MD-group had a history of anorexia that was remitted for several years at time of investigation. 14 patients had a recurrent depressive disorder with an average of around three (SD = 2.08) episodes and two had a first depressive episode. Participants were recruited from inpatient and outpatient units of the Department of Psychiatry and Psychotherapy III of the Ulm University Hospital. All 33 participants were right-handed according to the Edinburgh Handedness Inventory [40]. Regular smoking cigarettes was reported from three of the MD- and four participants of the HC-group but was prohibited at least 2 h before fMRI-scanning. Participants with any severe medical disorder, epilepsy, psychotic disorder, substance use disorder or regular consumption of alcohol or illegal drugs were excluded from the study. Antidepressant medication was not interrupted except of sedative drugs on the day of investigation. Patients of the MD-group took antidepressant medication of various kinds (see Supplementary material Table S1). One patient had a concomitant medication with topiramate and another patient with pregabalin, which were paused for a wash-out period of at least 3 days prior to fMRI-scanning (corresponding to 5 times the half-life of the substances). All participants gave written informed consent prior to the study that was approved by the local ethical committee of Ulm University and conducted in accordance with the Declaration of Helsinki.

**Table 1:** Demographical and behavioral data of healthy controls (HC) and patients with major depression (MD)

	HC	MD	*t*-test	
	Mean (sem)	Mean (sem)	*t*	*P*
*Demographical data*				
Age (years)	23.06 (1.03)	28.69 (1.15)	– 4.41	*0.001*
Education (school years)	10.82 (0.41)	10.81 (0.42)	0.02	0.985
*Smoking behavior*				
Subjects smoking cigarettes (*n*/total)	4/17	3/16		
Cigarettes per day	7 (8.98)	12 (9.85)	– 1.84	0.515
*Psychometric measures*				
BDI	3.24 (0.96)	33.63 (2.87)	– 11.86	*0.000*
BIS	57.76 (1.91)	65.25 (2.04)	– 3.25	*0.012*
*Incorrect incongruent NoGo trials*				
Errors of commission	13.24 (2.68)	7.75 (1.10)	2.36	0.074
Reaction time (ms)	420.63 (15.31)	452.28 (15.45)	– 1.77	0.156

Statistical analyses were conducted by two-sided unpaired samples t-tests. Significant results (*p* < 0.05) are highlighted in italic font

*BDI *beck depression inventory, *BIS *barratt impulsiveness scale, *sem *standard error of the mean, *ms *milliseconds

### Psychometric measurements

All participants were screened by using the Structured Clinical Interview for DSM-IV [SCID-I and -II; [41]] and clinical diagnoses of patients with MD were verified by one of the study psychologists or physicians. Current depressive symptoms were assessed by a German version of the Beck Depression Inventory, [second edition, BDI-II; [42]]. To assess impulsivity as personality trait, we used the Barratt Impulsiveness Scale 11th revision [BIS-11; [43–45]], a self-reporting questionnaire composed of 30 items, each rated on a 4-point Likert scale [44]. Higher total sum-scores reflect higher trait impulsivity. Two-sided *t*-tests for unpaired samples were computed to analyze psychometric scales.

### fMRI paradigm

**Fig. 1:**
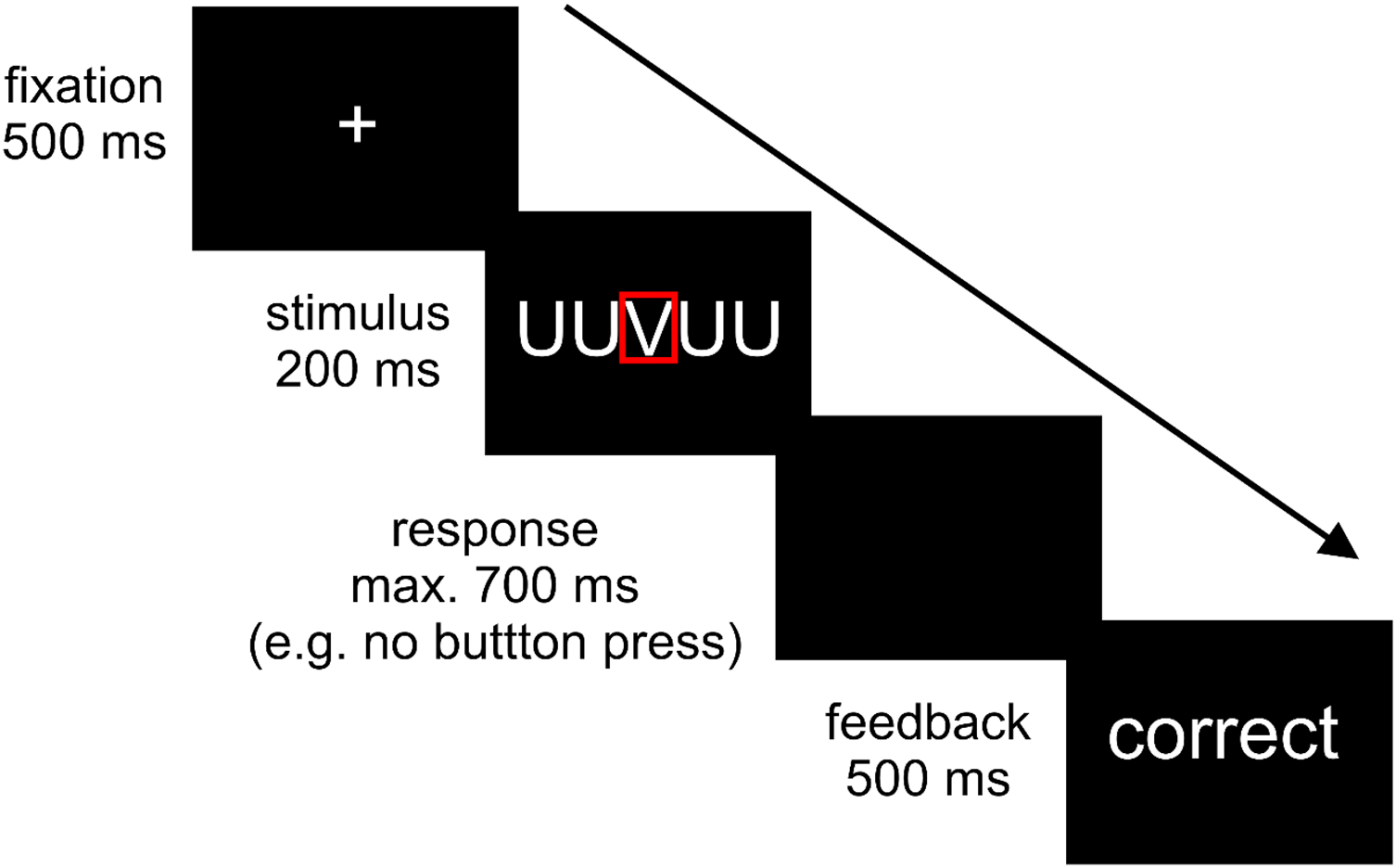
fMRI-task: Erikson-flanker Go/NoGo-paradigm. Presentation of each trial began with a centrally presented fixation cross for a period of 500 ms. Afterward, letter strings were centrally shown for a duration of 200 ms, followed by a blank screen with a duration of 700 ms. The trial ended with the presentation of feedback stimuli. According to the subjects´ performance, the German expressions for either correct or wrong were presented for 500 ms. The figure depicts a sequence of an incongruent NoGo trial. The target letter ‘V’ is highlighted by a red rectangle for demonstrational purposes

During fMRI, we used a combined Erikson-flanker Go/NoGo-paradigm [46], that has been established in several electrophysiological and fMRI studies on error processing [34–37]. Five-letter strings were presented comprising the letters R, U, P, and V, with the action-relevant target always mid-standing. During Go trials, subjects were asked to respond with their right index finger on a two-button box to the target letter R and with their right middle finger to the target letter U. In NoGo trials, subjects should withhold their response upon appearance of the letters P or V. Target and flanker stimuli were combined either congruently or incongruently. In congruent trials, all five letters were the same. In incongruent Go trials, targets were flanked by visually similar NoGo target letters (e.g., VVUVV). In incongruent NoGo trials, the central NoGo target was flanked by visually similar Go targets (e.g., UUVUU). The trial ended with the presentation of feedback stimuli (see Fig. 1). To ensure that task conditions and, in particular, incongruent NoGo trials provided enough errors for the study of error-related signals, the instruction to the participants emphasized speed over accuracy. Combination of the factors condition (Go, NoGo) and type (congruent, incongruent) yielded 66 trials of each level, resulting in a total of 264 trials. One trial lasted 1.9 s; mean intertrial interval was 3.01 s. The average stimulus-onset asynchrony for events of the same combination of condition by type (e.g., incongruent NoGo) was 19.5 s. The duration of the whole task was 22 min. Reaction times and correctness of subjects` responses on each trial were registered by a standard personal computer.

### fMRI data acquisition

Due to a scanner update during data acquisition, functional imaging data were obtained by a 3 T MAGNETOM Allegra (Siemens, Erlangen, Germany) for the HC-group, and by a 3 T MAGNETOM Prisma Scanner (Siemens, Erlangen, Germany) for the MD-group. A T2*-sensitive gradient echo sequence was used for functional imaging of both samples with an echotime (TE) of 33 ms, a flip angle of 90°, a field of view (FOV) of 230 mm, and a slice thickness of 2.5 mm with an interslice gap of 0.5 mm. At a repetition time (TR) of 2000 ms, 35 transversal slices were recorded with an image size of 64 × 64 pixels during the Go/NoGo task. High-resolution T1-weighted anatomical images were obtained using three-dimensional magnetization-prepared rapid acquisition with gradient echo sequences (1 × 1 × 1 mm voxels, band-width (BW) 130 Hz/ Pixel, TR 2500 ms, TI 1.1 s, echotime (TE) 4.57 ms, flip angle 12°).

### fMRI-data analysis

Image pre-processing and statistical analyses were carried out using Statistical Parametric Mapping (SPM12, Wellcome Department, London, UK) with a random effects model for group analyses. Data from each session were pre-processed including slice-timing, realignment and normalization into a standard template (Montreal Neurological Institute, MNI) with a spatial resolution of 2 × 2 × 2 mm^3^. Smoothing was applied with an 8-mm FWHM isotropic Gaussian kernel. Intrinsic autocorrelations were accounted for by AR (1) and low frequency drifts were removed via high-pass filtering (1/128 s). On the single subject level, individual event types were modeled as trains of delta functions at each stimulus onset convolved with a canonical hemodynamic response function. Fixed-effects analyses comprised modeling of individual events following the combination of the factors condition (Go, NoGo), type (congruent, incongruent) and response (correct, incorrect), resulting in eight conditions. The six realignment parameters were added to the design matrix.

According to our previous studies conducted with the same task with the primary focus on error processing [34, 37], second level group analyses were computed within a 2 × 2 analysis of variances (ANOVA) with the two factors ‘group’ (HC, MD) and ‘condition’ (NoGo/incongruent/correct, NoGo/incongruent/incorrect). Congruent NoGo trials did not consistently yield errors across subjects and were therefore not further analyzed. Computation of between-groups differences in neural activations related to errors of commission, were constrained to voxels significant (*p* < 0.05) within a conjunction analysis consisting of differential neural activation (incorrect minus correct incongruent NoGo trials) of each of the two groups. Within this inclusive mask, group differences were computed for the contrast of incorrect minus correct incongruent NoGo trials. Here, significant group differences were inferred at a statistical threshold of *p* < 0.001, uncorrected at the voxel level in combination with a minimum cluster size of 183 contiguously significant voxels, corresponding to an FWE-corrected *p*-value of *p* < 0.05 at the cluster level. This specific number of 183 voxels was computed with a script from Tom Nichols and Marko Wilke (CorrClusTh.m v1.12 2008/06/10) that defines the extent of a cluster of contiguously significant (e.g. *p* < 0.001) voxels, to survive a family-wise corrected cluster-size *p*-value of *p* < 0.05. The script can be found under https://warwick.ac.uk/fac/sci/statistics/staff/academic-research/nichols/scripts/spm/johnsgems5/#Gem6. Due to fMRI data acquisition on two different MRI-scanners and significant differences in age of the two groups (MD and HC), scanner type and age were included as covariates in all fMRI-analyses. We also visually inspected one randomly selected EPI volume per each subject obtained from the Siemens MAGNETOM Allegra and the Siemens MANGETOM Prisma scanner, and found no indication for systematic differences in image quality. Moreover, only effects of condition differences but not of single conditions were allowed to enter computation group differences for statistical inference since only condition differences would compensate for putative, systematic contrast-to-noise scanner differences.

In case of significant group-by-condition interactions, individual differential neural activations (incorrect NoGo minus correct NoGo trials; averaged over significant voxels) in the MD-group only were correlated with individual numbers of errors, BDI and BIS scores by computing Pearson correlation coefficients.

## Results

### Demographical and behavioral data

In line with the clinical diagnosis, significantly higher BDI sum-scores were observed in patients with MD relative to HC. In addition, we observed significantly higher BIS sum-scores, measuring impulsivity as personality trait, in MD compared to HC. Regarding the behavioral responses in the Go/NoGo task during fMRI, reaction times and number of errors for incorrect incongruent NoGo trials did not significantly differ between MD and HC. Corresponding mean scores, *t*- and *p*-values of the psychometric measurements and task responses in NoGo trials during fMRI are summarized in Table 1. In addition, we observed significant slower reaction times in correct Go trials in MD relative to HC, whereas the number of correct responses in congruent and incongruent Go trials did not differ significantly between groups. Details on task responses in Go trials are provided in our supplementary material section (see Table S2).

### fMRI data

A conjunction analysis comprising differential neural activations contrasting incorrect versus correct incongruent NoGo trials in each group, MD and HC, revealed significant (*p* < 0.05; FWE-corrected on cluster level) differential neural activations within the right and left anterior insula, the right and left inferior frontal gyrus (IFG), the left supramarginal gyrus, the left dACC and the adjacent pre-SMA and thus, in brain regions that have been consistently associated to neural error processing. More details are provided in our supplemenatry material section (see Table S3 and Fig. S1).

**Fig. 2:**
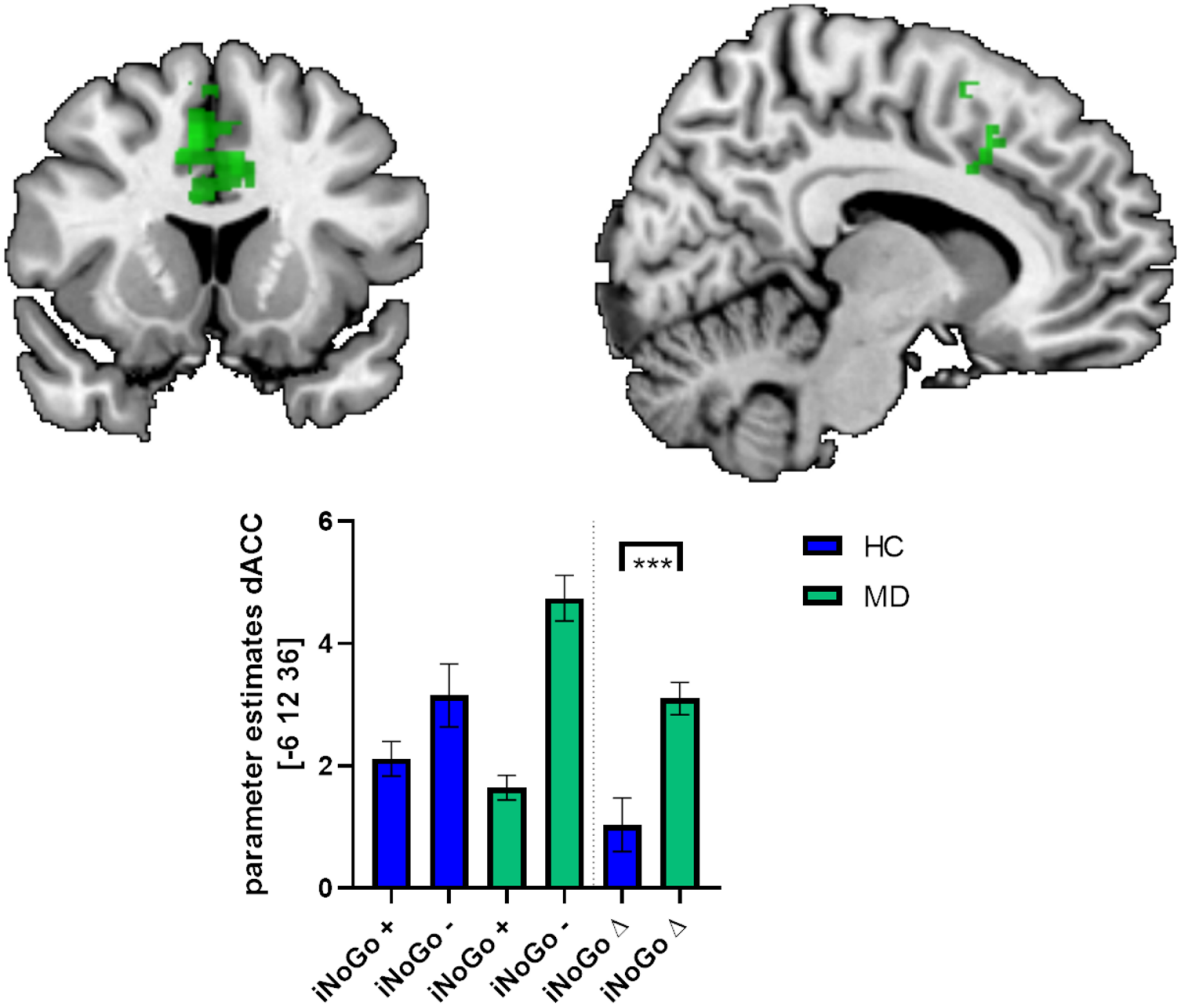
Significant differential (incorrect minus correct incongruent NoGo-trials) neural activations corresponding to errors of commission in patients with major depression (MD; *n* = 16) compared to healthy controls (HC; *n* = 17) masked within a conjunction analyses of differential (incorrect minus correct incruent NoGo trials) neural activations in HC and MD at a statistical threshold of *p* < 0.05. Bar charts depict fMRI parameter estimates extracted from the significant cluster within the dorsal anterior cingulate cortex (dACC) and the pre-supplementary motor area (pre-SMA) for HC and MD; error bars is standard error of the mean. *HC *healthy controls, *MD *patients with major depression; iNoGo +  = correct incongruent NoGo-trials; *iNoGo *incorrect incongruent NoGo-trials; *iNoGo* ∆ incorrect minus correct incongruent NoGo-trials; *** = statistical significance (*p* < 0.05, FWE-corrected on cluster level)

Comparing both groups, we observed significant (*p* < 0.05; FWE-corrected on cluster level) higher differential (incorrect versus correct incongruent NoGo trials) neural activations due to commission errors in MD relative to HC within pre-SMA and dACC (see Table 2 and Fig. 2). No significantly higher differential neural activations were observed in HC compared to MD.

**Table 2:** Significant (*p* < 0.001, k > 183Vx; FWE-corrected on cluster level) differential (incorrect minus correct incongruent NoGo trials) neural activations in patients with major depression (MD; *n* = 16) compared to healthy controls (HC; *n* = 17), with scanner type and age as covariates

	BA	Anatomic	L/R	Cluster size	Z	MNI		
		Label				x	Y	Z
MD > HC	6	Pre-SMA	R	358	4.64	8	14	44
			R		4.21	8	6	56
	24	dACC	L		4.21	-6	12	36

*BA *Brodman area, *L *left, *R *right, *MNI *montreal neurological insitute (x-, y-, z-coordinates are provided in mm); *Z *z-score of standard norm distribution, *dACC *dorsal anterior cingulate cortex, pre-SMA pre-supplementary motor area

### Correlation analyses

No significant correlations between individual numbers of errors, BDI and BIS scores and individual differential neural activations during commission errors were observed in the MD-group.

## Discussion

Several electrophysiological and few fMRI studies have investigated neurofunctional alterations of error processing in MD. However, corresponding results are far from consistent. We investigated a sample of young female patients with MD and HC with fMRI and an established Erikson-flanker Go/NoGo-paradigm, to support reliable evidence for a neurofunctional signature of error processing in MD. Despite a comparable number of commission errors in MD relative to HC, we observed a significant increase in error-related neural activations in MD within the dACC and the adjacent pre-SMA and thus in brain regions that were consistently associated to neural error processing.

According to the clinical diagnoses and similar to other studies [47–49], we observed significant higher BDI and BIS sum-scores in MD relative to HC. During the Erikson-flanker Go/NoGo-paradigm, the rate of commission errors in incongruent NoGo trials did not significantly differ between MD and HC. An equal or even a fewer rate of commission errors in MD is in line with previous observations [10, 24–26, 50] and has been interpreted as a cause of psychomotor retardation on the one hand, or a trade-off between accuracy and speed in MD on the other, in which responses are slower and perhaps more careful. Our finding of slower reaction times in correct and incorrect Go trials in MD and, although not statistically significant, slower reaction times and lower error rates in MD relative to HC in incongruent NoGo trials, is in line with this interpretation. Of note, a comparable rate of errors in both groups (MD and HC) may strengthen the interpretability of our fMRI results considering that neural alterations may not be confounded by an increased error rate [51].

During fMRI, we observed differential neurofunctional activations related to commission errors within the dACC, pre-SMA, right and left IFG and anterior insula, as well as left supramarginal gyrus in HC and MD. Enhanced neural activations of these brain regions have been consistently associated with error processing [11, 12, 15–18, 52], supporting that our task induced reliable neural responses.

Comparing the two groups, MD and HC, we observed significantly enhanced differential neural activations related to errors of commission within the dACC and the pre-SMA in MD relative to HC. The dACC responds to various types of performance feedback such as errors or conflicts [52]. Increased neural activations within this region were consistently associated with performance and error monitoring [13, 15, 16, 53, 54]. Beyond a pure monitoring function, the dACC also directs attention toward task-relevant stimuli and maintains associations between actions and their outcomes, including the implementation of task sets [52]. The neural signaling of the dACC may serve to improve task performance by modulating control over the motor system and allocations of capacities of different competing neural systems [9]. Our observation of enhanced neural activations within the dACC during commission errors in MD is also in line with several electrophysiological and one fMRI study [19–21, 29], that demonstrated an increased neural response pattern of this region during error processing in MD. By contrast, two other fMRI studies described diminished error-related neural activations of frontal brain areas including the ACC [27, 28]. It is of note, however, that both studies investigated patients with remitted depressive disorder rather than patients with current MD as in our study. In addition, we observed significantly enhanced neural activations of the pre-SMA. Neural activations of this region have been associated with error detection [6, 8, 13, 59] and the identification of response conflicts [16, 54, 60, 61], with errors representing a special case of higher order conflict processing [13, 60]. Beyond the observation that both, dACC and pre-SMA contribute to the ERN [11–14], there is evidence that these two regions response at different points in time. In particular, neural activations of the pre-SMA due to errors are thought to emerge earlier and to provide [62] and to precede [63] error-related signals as an input to the dACC. It has been also suggested that the pre-SMA reliably responds to negatively valenced signals that may arise from either error or conflict monitoring. In addition, neural signaling within the pre-SMA encodes the information to evaluate previous decision processes, necessary to learn from errors and to adjust erroneous behavior [64]. Unfortunately, we were not able to specifically investigate post-error adjustments as trial composition and stimulus arrays in our task are inappropriate for this purpose.

With enhanced neural activations in error-related brain regions in the absence of statistically significant differences in error rates in MD compared to HC, our data support an increased responsivity of neural error detection in MD compared to HC. These enhanced neural responses may reflect a potential neural correlate of the clinically relevant and frequently observed enhanced sensitivity to negative environmental cues or errors [3–5]. However, a growing body of work indicates that also substance abuse is characterized by abnormal error-related neural responses of the ACC [55–58], potentially related to or mediated by increased impulsivity. This speaks against clinical specificity of our present observation. On the other hand, patients with substance disorder were not included in our study, and we also did not find a significant correlation between differential dACC/pre-SMA activations and individual BIS scores in MD.

Some shortcomings are to discuss. Our study was conducted in a relatively small sample size comprising only female participants. This may compromise the strength and the generalizability of our data, and present results await empirical replication with larger samples of both sexes. Moreover, our patients with MD were investigated under antidepressant medication that may have potentially altered neural activations relevant for error processing. However, similar error-related neural alterations in MD were also found in patients with MD without antidepressant medication [10]. Also, another electrophysiological study observed no differences in error-/event-related potential amplitudes between patients with MD with and without antidepressant medication [65]. As another limitation, mean age of patients with MD and controls differed significantly by about 5 years and patients were investigated with a different fMRI-scanner of the same manufacturer with the same field strength and acquisition parameter as HC. For compensation, age and scanner type were included as covariates in all fMRI-analyses. Also, visual inspection focusing on systematic differences in image quality between both MR scanners revealed no evidence that would speak against the comparability of fMRI activation patterns derived from both devices. We also found no hints for considerable differences in contrast-to-noise ratios between the two scanners, which is supported by the rather equivalence of neural signaling during incongruent correct NoGo trials in both groups as indicated in our Fig. 2. Finally, even if one MR scanner would have had a systematically greater contrast-to-noise ratio than the other, this signal gain would have affected both event types (correct and incorrect NoGo trials). Since the critical dependent variables were never signal changes against baseline but always signal changes between trials of different event types, any putative global signal differences are automatically taken into account by these condition differences used for between-group comparisons in our analysis.

## Conclusion

The neural system for the identification of errors has been intensively investigated in healthy subjects by electrophysiology and functional neuroimaging studies. In major depression, the clinical observation of increased sensitivity to errors and negative feedback may suggest alterations in neural networks for error processing. However, reliable evidence for these neural alterations is limited by inconsistent findings despite their relevance as potential neurobiological marker of clinically relevant psychopathology. We therefore investigated a sample of female patients with current major depression by fMRI and an Erikson-flanker Go/NoGo-paradigm compared to HC. In the absence of significant differences in error rates of commission, we observed significantly enhanced neural activations of the dACC and the pre-SMA in MD relative to HC. These brain regions are well related to neural error processing. Present results therefore support the notion of enhanced responsivity of neural error processing mechanisms in MD as a potential neural signature of this disorder. Whether enhanced error-related recruitment of the dACC and pre-SMA is merely an expression of increased negative feedback responsibility of patients with depression or whether this phenomenon is functionally in service of ongoing behavioral adjustments, successful or unsuccessful, cannot be answered with present data and remains an open question for future studies in search of clinically relevant imaging biomarkers of depression.

## Supplementary Information

Below is the link to the electronic supplementary material.

Supplementary file1 (DOCX 434 KB)
