# Supplementary material for: Neural signature of error processing in major depression
Source: Eur Arch Psychiatry Clin Neurosci. 2021 Feb 17;271(7):1359–68. doi: 10.1007/s00406-021-01238-y (PMC8429380; doi:10.1007/s00406-021-01238-y)

**Supplementary Material**

**Neural Signature of Error Processing in Major Depression**

Kathrin Malejko^1*^, Stefan Hafner^1^, Paul L. Plener^2,3^, Martina Bonenberger^2^, Georg Groen^1^, Birgit Abler^1^, Heiko Graf^1^

^1^ Department of Psychiatry and Psychotherapy III, University Hospital Ulm, Germany

^2^ Department of Child and Adolescent Psychiatry and Psychotherapy, University Hospital Ulm, Germany

^3^ Department for Child and Adolescent Psychiatry, Medical University of Vienna, Vienna, Austria

**Table S1:** Medication in patients with major depression (MD; n=16). None of the healthy controls (HC; n=17) took any medication.

| *Medication* | *MD* |
| --- | --- |
| Sertraline | 6 |
| Venlafaxine | 2 |
| Escitalopram | 2 |
| sertraline + mirtazapine | 2 |
| Fluoxetine | 1 |
| Bupropion | 1 |
| bupropion + escitalopram | 1 |
| fluoxetine + mirtazapine | 1 |

|  | HC | MD | t-test | |
| --- | --- | --- | --- | --- |
|  | mean (sem) | mean (sem) | t | P |
| *Number of correct congruent Go trials* | 23.06 (2.75) | 29.75 (3.20) | -1.92 | 0.121 |
| reaction time (in ms) | 396.99 (9.20) | 447.28 (9.57) | -4.59 | *0.001* |
| *Number of correct incongruent Go trials* | 20.24 (2.49) | 25.44 (3.00) | -1.61 | 0.190 |
| reaction time (in ms) | 399.28 (11.45) | 449.86 (9.51) | -3.95 | *0.002* |

**Table S2:** Task responses in Go trials during fMRI in the Erikson-flanker Go/NoGo-paradigm of patients with major depression (MD) and healthy controls (HC). Statistical analyses were conducted by two-sided unpaired two-sample t-tests. Significant results (p<0.05) are highlighted in italic font.

ms=milliseconds, sem=standard error of the mean

**Table S3:** Significant (p<0.001, k>183 continguously significant voxels; corresponding to p<0.05, FWE-corrected on cluster level) neural activations in the conjunction analysis of the differential (incorrect minus correct incongruent NoGo trials) contrast in healthy controls (HC; n=17) and in patients with major depression (MD; n=16).

| *BA* | *Anatomic label* | *L/R* | *cluster size* | *Z* | *MNI* | | |
| --- | --- | --- | --- | --- | --- | --- | --- |
|  |  |  |  |  | *x* | *y* | *z* |
| 24 | dACC | L | 471 | 3.91 | -6 | 20 | 26 |
| 6 | pre-SMA |  |  | 4.24 | -8 | 20 | 54 |
| 48 | inferior frontal gyrus | R | 1084 | 5.44 | 50 | 14 | 2 |
|  | anterior insula |  |  | 4.78 | 36 | 16 | -10 |
| 48 | inferior frontal gyrus | L | 840 | 4.61 | -50 | 14 | -2 |
|  | anterior insula |  |  | 5.20 | -32 | 20 | -4 |
| 40 | gyrus supramarginalis | L | 326 | 4.09 | -54 | -26 | 32 |

BA=Brodman area; L=left; R=right; MNI=Montreal Neurological Insitute (x-, y-, z-coordinates are provided in mm), Z=z-score of standard norm distribution; dACC=dorsal anterior cingulate cortex; pre-SMA=pre-supplementary motor area.

**Figure S1:** Significant (p<0.001, k>183 continguously signficacnt voxels; corresponding to p<0.05, FWE-corrected on cluster level) neural activations in the conjunction analysis comprising neural activations during incorrect (iNoGo -) minus correct (iNoGo +) responses during incongruent NoGo trials in healthy controls (HC; n=17) and patients with major depression (MD; n=16).

Bar charts depict fMRI parameter estimates extracted from the significant clusters within the dorsal anterior cingulate cortex (dACC) and right and left inferior frontal gyrus (IFG) for HC and MD; errors bars depict standard error of the mean.


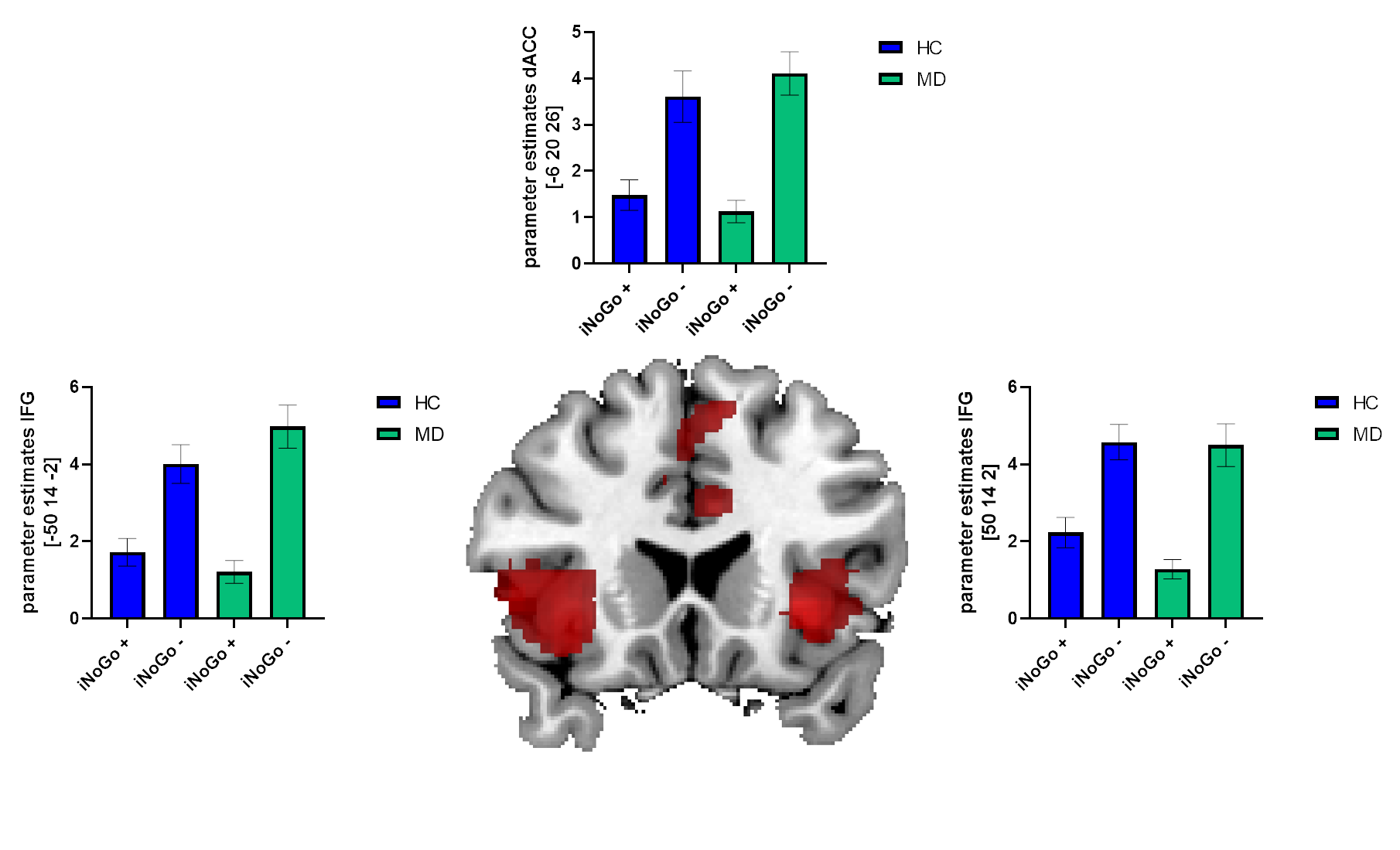

Supplement: Supplementary file 1 — Supplementary file1 (DOCX 434 KB) [file 406_2021_1238_MOESM1_ESM.docx]
